# Investigating Microstructural Changes in White Matter in Multiple Sclerosis: A Systematic Review and Meta-Analysis of Neurite Orientation Dispersion and Density Imaging

**DOI:** 10.3390/brainsci11091151

**Published:** 2021-08-29

**Authors:** Abdulmajeed Alotaibi, Anna Podlasek, Amjad AlTokhis, Ali Aldhebaib, Rob A. Dineen, Cris S. Constantinescu

**Affiliations:** 1 Department of Radiological Sciences, School of Applied Medical Sciences, King Saud bin Abdul-Aziz University for Health Sciences, Riyadh 14611, Saudi Arabia; aldhebaiba@gmail.com; 2 Division of Clinical Neuroscience, School of Medicine, Nottingham University Hospitals NHS Trust, University of Nottingham, Nottingham NG7 2UH, UK; Anna.Podlasek@nottingham.ac.uk (A.P.); amjad.altokhis@nottingham.ac.uk (A.A.); Rob.Dineen@nottingham.ac.uk (R.A.D.); Cris.Constantinescu@nottingham.ac.uk (C.S.C.); 3 NIHR Nottingham Biomedical Research Centre, Nottingham NG1 5DU, UK; 4 Department of Radiological Sciences, School of Health and Rehabilitation Sciences, Princess Nourah Bint Abdulrahman University, Riyadh 11564, Saudi Arabia; 5 Department of Neurology, Cooper University Hospital, Camden, NJ 08103, USA

**Keywords:** neurite orientation dispersion and density imaging, NODDI, multi-shell diffusion, multi-compartment diffusion, multiple sclerosis, MS

## Abstract

Multiple sclerosis (MS) is characterised by widespread damage of the central nervous system that includes alterations in normal-appearing white matter (NAWM) and demyelinating white matter (WM) lesions. Neurite orientation dispersion and density imaging (NODDI) has been proposed to provide a precise characterisation of WM microstructures. NODDI maps can be calculated for the Neurite Density Index (NDI) and Orientation Dispersion Index (ODI), which estimate orientation dispersion and neurite density. Although NODDI has not been widely applied in MS, this technique is promising in investigating the complexity of MS pathology, as it is more specific than diffusion tensor imaging (DTI) in capturing microstructural alterations. We conducted a meta-analysis of studies using NODDI metrics to assess brain microstructural changes and neuroaxonal pathology in WM lesions and NAWM in patients with MS. Three reviewers conducted a literature search of four electronic databases. We performed a random-effect meta-analysis and the extent of between-study heterogeneity was assessed with the I2 statistic. Funnel plots and Egger’s tests were used to assess publication bias. We identified seven studies analysing 374 participants (202 MS and 172 controls). The NDI in WM lesions and NAWM were significantly reduced compared to healthy WM and the standardised mean difference of each was −3.08 (95%CI −4.22 to (−1.95), *p* ≤ 0.00001, I2 = 88%) and −0.70 (95%CI −0.99 to (−0.40), *p* ≤ 0.00001, I2 = 35%), respectively. There was no statistically significant difference of the ODI in MS WM lesions and NAWM compared to healthy controls. This systematic review and meta-analysis confirmed that the NDI is significantly reduced in MS lesions and NAWM than in WM from healthy participants, corresponding to reduced intracellular signal fraction, which may reflect underlying damage or loss of neurites.

## 1. Introduction

Multiple sclerosis (MS) is an autoimmune disease that affects almost 2.5 million individuals worldwide, often in young adulthood [1] MS is characterised by widespread damage of the central nervous system that includes alterations in normal-appearing white matter (NAWM) and demyelinating white matter (WM) lesions [2].

Magnetic resonance imaging (MRI) plays an essential role in diagnosing and monitoring the disease course and the treatment effectiveness in MS. However, conventional MRI techniques have limited sensitivity to quantify microstructural alterations accompanying neuroaxonal degeneration in brain WM and MS lesions [3,4,5]. Quantitative MRI biomarkers of the brain provide a more sensitive detection of axonal degeneration and may become a more reliable outcome measure [6,7]. Diffusion tensor imaging (DTI) is a quantitative technique that has been widely used to characterise the microstructural abnormalities within both NAWM and WM lesions in MS [8]. Many studies have reported reduced fractional anisotropy (FA) and increased mean diffusivity (MD) in NAWM in MS patients compared with healthy participants [9,10,11,12] and one meta-analysis has confirmed a significant reduction of FA that suggested a widespread WM damage in MS [13]. Although these abnormalities may occur early in MS [14], DTI changes in the NAWM in patients with MS are linked with significant disability [15]. MRI-histopathological studies have confirmed a high correlation between the DTI changes and axonal count in WM lesions and NAWM, indicating that these abnormalities may reflect pathological alterations related to disability [16,17,18]. Despite the DTI sensitivity in detecting microstructural changes in WM, the lack of specificity is one of the caveats of DTI. In particular, DTI indices are affected by the orientation dispersion of fibres [19], which may lead to misinterpretation [20]. Furthermore, the interpretation of DTI parameters becomes more complex when two or more different tissues with diffusion properties are present in a single voxel [20].

A biophysical diffusion model known as neurite orientation dispersion and density imaging (NODDI) has been proposed to overcome some DTI limitations, providing a more precise characterization of WM microstructures [21]. NODDI describes brain tissue as a simplified combination of three compartments. A Watson distribution assumption of sticks models the first compartment, known as the intracellular compartment (axons and dendrites), and the signal of each stick is assumed to be a degenerated diffusion tensor with a perpendicular diffusivity equal to zero. The extracellular compartment is a Gaussian anisotropic diffusion, as seen in DTI, and the free water compartment is an isotropic Gaussian diffusion such as Cerebral Spinal Fluid (CSF). After fitting the NODDI model, maps can be calculated for the Neurite Density Index (NDI), the Orientation Dispersion Index (ODI), and isotropic signal fraction (isoVF). These maps explicitly estimate the orientation dispersion and neurite density, all of which contribute to conventional DTI parameters such as FA [20]. Although NODDI has not been widely applied in MS, this technique is promising in investigating the complexity of MS pathology, as it is more specific than DTI in detecting microstructural alterations [20,22,23,24,25]. Recent studies found that DTI and myelin-sensitive imaging are less sensitive than NODDI to neurite density and changes in NAWM and WM lesions in patients with MS [23,24,26]. In addition, changes in NODDI parameters in specific brain WM [22,23], such as the internal capsule and corpus callosum in which long-tract axons are found [27], are associated with disability [22,23]. However, some other studies have unexpectedly reported increased fibre coherence (as shown by the low ODI) in WM lesions and NAWM compared to healthy WM, which suggests careful interpretation in the presence of severe axonal loss (as indicated by low NDI) [20,24,25,26]. Unfortunately, longitudinal studies, which may help clarify the relationship between disease activity/progression and NODDI-detected WM changes, are absent in the literature.

To summarise the current literature in this area, we conducted a systematic review and meta-analysis of studies using NODDI metrics to assess brain microstructural changes and neuroaxonal pathology in WM lesions and NAWM in patients with MS.

## 2. Materials and Methods

### 2.1. Study Registration

This systematic review and meta-analysis were conducted according to the Preferred Reporting Items for Systematic Reviews and Meta-Analysis (PRISMA) guidelines [28] and registered in the International Prospective Register of Systematic Reviews (PROSPERO) database (CRD42021239169) in February 2021.

### 2.2. Study Selection (Inclusion and Exclusion Criteria)

Studies were considered for inclusion if they: (i) used a cross-sectional and cohort design; (ii) were published in peer-review journals; (iii) were written in English; (iv) reported NODDI coordinates (NDI and ODI) of NAWM and WM lesions in people with MS compared to healthy WM at 3.0 T MRI; (v) included participants aged 18–75 years old; (vi) included healthy controls with no neurological disorders; (vii) included MS participants who could have any MS sub-types; or (viii) investigated the brain microstructural alterations in WM MS lesions and NAWM. Studies were considered for exclusion if they: (i) were published as reviews or systematic reviews, qualitative studies, opinion pieces, editorials, comments, technical or validation/reproducibility studies, or were published as not full-text articles; (ii) reported subjects with neurological disorders and other diseases not due to MS; or (iii) reported results on animals, in-vitro, or MS patients only. Before the review registration, all inclusion/exclusion criteria were reviewed carefully by an experienced neurologist (C.S.C) and neuroradiologist (R.A.D).

### 2.3. Sources, Search Strategy and Screening

Four electronic databases were searched (Medline (Northfield, IL, USA), Embase (Amsterdam, Netherlands),Scopus (Amsterdam, Netherlands), and PubMed (Bethesda, Maryland, USA)) with the keywords (“Neurite Orientation Dispersion and Density Imaging” or “NODDI”, “Multi-shell Diffusion”, “Multi-compartment Diffusion”and “Multiple Sclerosis” or “MS”). Three subspecialised reviewers, namely A.A, A.D, and A.T., conducted the search strategy, database selections, study screening/identification, study eligibility/inclusion, and quality assessment independently and blindly. Citation chaining from reviews and other papers discovered by reviewers was carried out and the searches were re-run before the final analysis in April 2021. Mutual discussions solved disagreements between reviewers.

### 2.4. Data Extraction and Collection

In March 2021, the first author A.A. extracted and organised the desired data from all included studies using Microsoft Excel. The demographics of the MS, control groups, and clinical features of MS patients were extracted (age, gender, disease duration, EDSS score, population groups, and sample size). The between-group findings based on the region of interest analysis (ROI) were extracted and organised for analysis (mean/standard deviation (SD) of the NDI and ODI values in WM lesions, NAWM, and healthy WM). For the data synthesis, corresponding authors from the identified studies were emailed for a maximum of three times, separated by one week, to determine their willingness to contribute the raw data. Requested data included the SD and mean of the NDI and ODI values for MS lesions and NAWM per subject, along with corresponding Expanded Disability Status Scale (EDSS) and MS duration.

### 2.5. Outcome Measures

The primary outcome measure was assessing the Neurite Density Index (NDI) and it was used to quantify microstructural abnormalities in neuroaxonal pathology (loss of axonal density or integrity) in WM MS lesions and NAWM compared with the WM in healthy controls. The secondary outcome measure assessed the Orientation Dispersion Index (ODI), which was used to quantify the loss of fibre coherence (an increase in fibre dispersion) in NAWM and WM MS lesions compared with the WM in healthy controls.

### 2.6. Statistical Analysis

Study characteristics and extracted variables were summarized using standard descriptive statistics. Continuous variables were expressed as means and SD, and categorical variables were expressed as frequencies or percentages. The continuous variables in this meta-analysis are presented as standardised mean differences (MD) with a 95% CI. A random-effects model and the Mantel–Haenszel method were used. Heterogeneity tests were conducted with the Q statistic distributed as a chi-square variate (assumption of homogeneity of effect sizes). The I^2^ statistic was used to assess the extent of between-study heterogeneity. Study heterogeneity I^2^ values > 50% were considered substantial and >75% were deemed considerable heterogeneity. Funnel plots and Egger’s tests were used to assess publication bias for the primary outcome. The Quadas-2 tool was used to evaluate the individual risk of bias in each study. P-values were two-tailed with values < 0.05 considered statistically significant. JASP 0.14.1.0 (University of Amsterdam, Amsterdam, Netherlands) and Review Manager 5.4.1 software (Cochrane organisation, London, UK) were used to implement all analyses.

## 3. Results

### 3.1. Included Studies and Sample Characteristics

**Figure 1:**
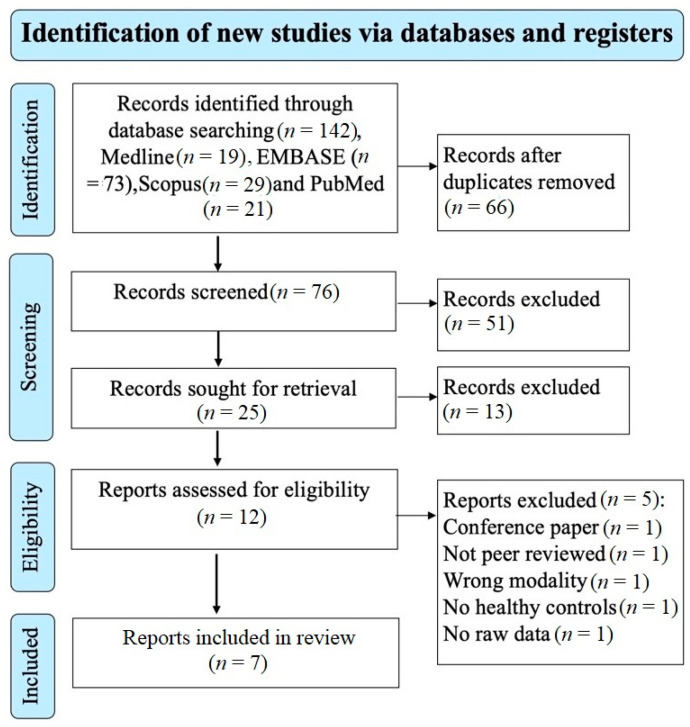
Prisma flow diagram showing the systematic search strategy, study identification, screening, and eligibility and inclusion criteria.

The electronic search of the four databases retrieved 142 studies. A total of 76 studies remained after removing the duplicate studies and screening the title and abstract excluded 51 studies that did not meet the eligibility criteria. A total of 25 studies remained for retrieval; 13 studies were excluded. 12 studies remained for the eligibility assessment; 5 studies were excluded based on the reasons shown on the PRISMA diagram (Figure 1). A total of 7 studies investigating the brain microstructure in patients with MS using NODDI were included and met the inclusion/exclusion criteria for this meta-analysis [20,23,24,25,26,27,29]. A total of 202 patients with MS (mean age of 40.1 years) and 172 healthy controls (mean age of 35.1 years) were included. The mean MS duration of all the studies was 8.3 years; however, two studies did not report disease duration [26,29]. There was no significant difference in the age and sex distribution between MS patients and healthy controls, with the caveat that one study did not provide information relating to the sex of participants [25]. In total, 125 of the included participants with MS were on treatment (diemtyhyl fumarate = 24, glatiramer acetate = 11, interferon-beta 1a = 10, natalizumab = 5, glucocorticoids = 6, glucocorticoids and on unspecified disease-modifying treatment = 1, unspecified disease-modifying therapy and no glucocorticoids, mitoxantrone = 1, fingolimod = 5, rituximab = 10, and ocrelizumab = 49). Additional clinical and technical characteristics are summarised in Table 1 and Table 2.

### 3.2. NDI in MS WM Lesions (MS Subjects) vs. Controls

**Figure 2:**
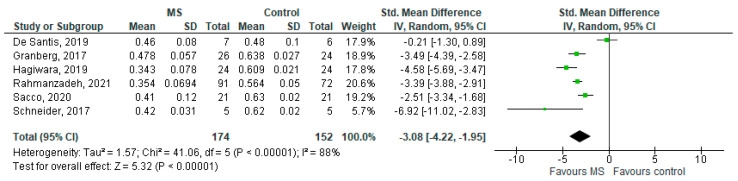
Forest plot for the standardised mean difference of the NDI in MS WM lesions (MS vs. control) [20,23,24,25,26,29]. Abbreviations: SD = standard deviation; CI = confidence interval; NDI = Neurite Density Index; MS = multiple sclerosis; WM = white mater; vs. = versus; and I2 = Heterogeneity Index.

**Figure 3:**
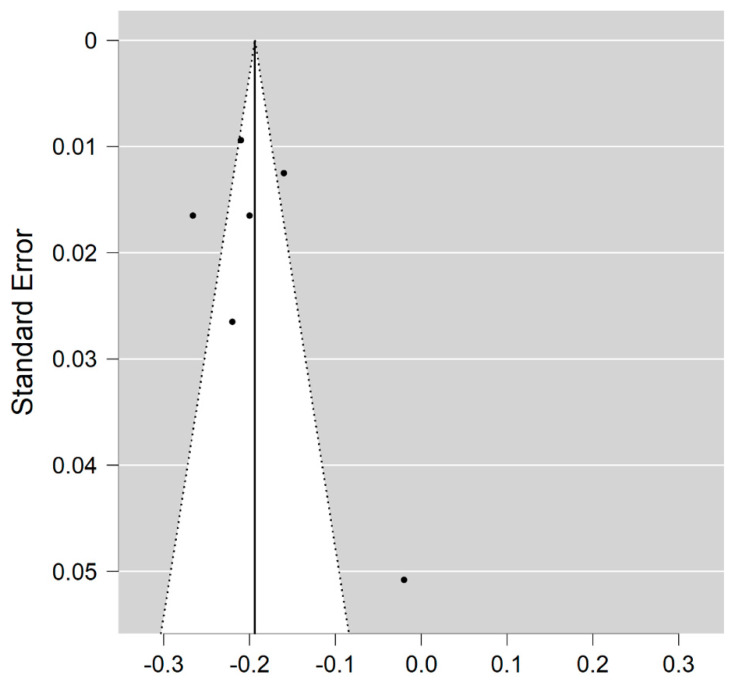
Funnel plot for the NDI in MS WM lesions (MS vs. control).

Six studies reported on the NDI and on WM lesions among 174 MS patients and 152 healthy controls [20,23,24,25,26,29]. The standardised mean difference was −3.08 (95% CI −4.22 to (−1.95)), *p* ≤ 0.00001 (Figure 2). There is considerable heterogeneity between studies I^2^ = 88%. The Egger’s test was *z* = 2.135, *p* = 0.033, which suggests potential publication bias (Figure 3).

### 3.3. NDI in NAWM (MS Subjects) vs. Controls

**Figure 4:**
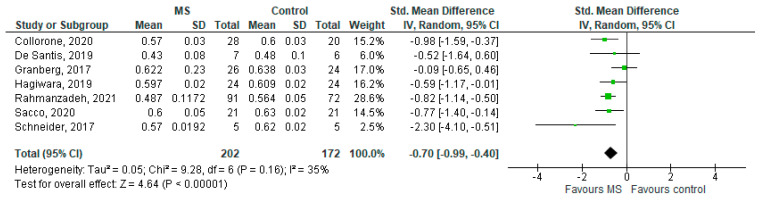
Forest plot for the standardized mean difference of the NDI in NAWM (MS vs. control) [20,23,24,25,26,27,29]. Abbreviations: SD = standard deviation; CI = confidence interval; NDI = Neurite Density Index; MS = multiple sclerosis; NAWM = normal-appearing white matter; vs. = versus; and I2 = Heterogeneity Index.

**Figure 5:**
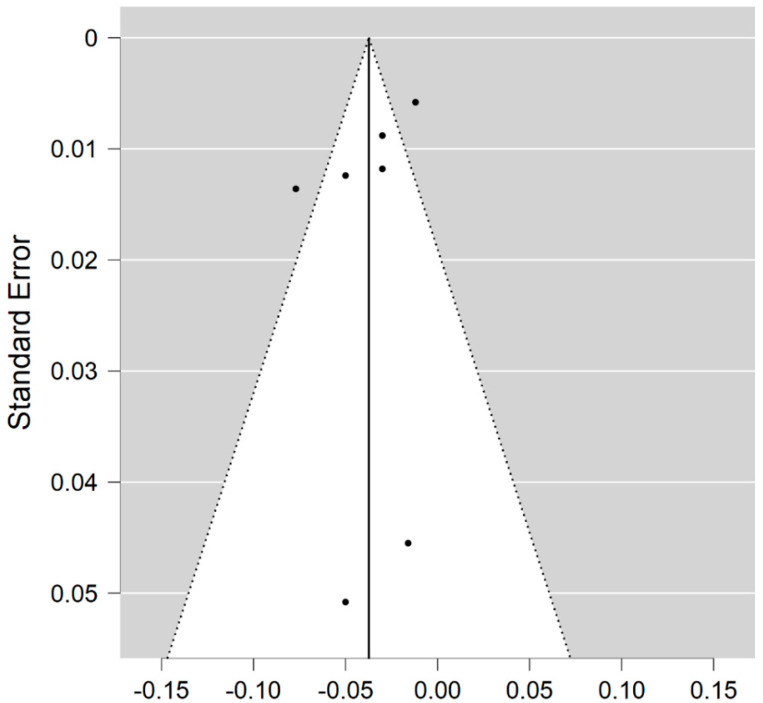
Funnel plot for the NDI in NAWM (MS vs. control).

Seven studies reported on the NDI and on the NAWM among 202 MS patients and 172 healthy controls [20,23,24,25,26,27,29]. The standardised mean difference was −0.70 (95% CI −0.99 to (−0.40)), *p* ≤ 0.00001 (Figure 4). Thus, there is moderate heterogeneity between studies I^2^ = 35%. The Egger’s test was *z* = −0.377, *p* = 0.706, which suggests no publication bias (Figure 5).

### 3.4. ODI in MS WM Lesions (MS Subjects) vs. Controls

**Figure 6:**
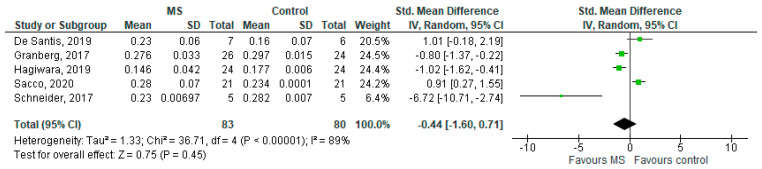
Forest plot for the standardised mean difference of the ODI in MS WM lesions (MS vs. control) [20,23,24,25,26]. Abbreviations: SD = standard deviation; CI = confidence interval; ODI = Orientation Dispersion Index; MS = multiple sclerosis; WM = white mater; vs. = versus; and I2 = Heterogeneity Index.

Five studies reported on the ODI and on MS WM lesions among 83 MS patients and 80 healthy controls [20,23,24,25,26]. The standardised mean difference was −0.44 (95% CI −1.60 to (−0.71)), *p* = 0.45. There is considerable heterogeneity between studies as I^2^ = 89% (Figure 6).

### 3.5. ODI in NAWM (MS Subjects) vs. Controls

**Figure 7:**
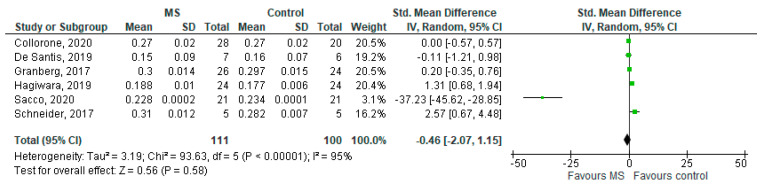
Forest plot for the standard mean difference of the ODI in NAWM (MS vs. control) [20,23,24,25,26,27]. Abbreviations: SD = standard deviation; CI = confidence interval; ODI = Orientation Dispersion Index; MS = multiple sclerosis; NAWM = normal-appearing white matter; vs. = versus; and I2 = Heterogeneity Index.

Six studies reported on the ODI and on the NAWM among 111 MS patients and 100 healthy controls [20,23,24,25,26,27]. The standardised mean difference was −0.46 (95% CI −2.07 to (−1.15)), *p* = 0.58. Thus, there is considerable heterogeneity between studies as I^2^ = 95% (Figure 7).

## 4. Discussion

This systematic review of published NODDI studies comparing NODDI metrics from lesions and NAWM in MS with the WM in healthy controls identified sufficient studies to allow for meta-analysis.

**Figure 8:**
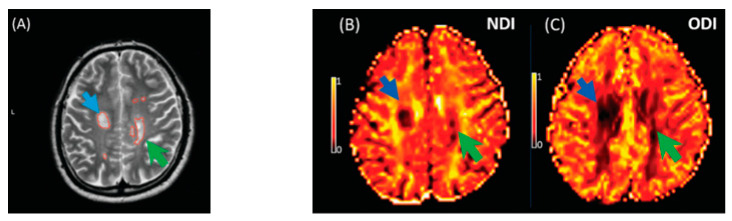
Illustrates NODDI metrics in a single slice of one MS subject. The MS lesion in the major white matter tracts (blue arrow) and periventricular lesion (green arrow) are marked in a structural MRI image (**A**), NDI map (**B**), and ODI map (**C**). Both the NDI and ODI are decreased in the MS lesion [20].

The key finding of this meta-analysis of ROI data concerned that the NDI was reduced in MS lesions and NAWM compared to healthy controls (Figure 8). This significant reduction of the NDI corresponds to reduced intracellular signal fraction and may reflect underlying damage or loss of neurites. Previous work has shown that this reduction is accompanied by higher MD and lower FA [24], and the higher ODI suggests demyelination, axonal loss, and less coherence in the fibre orientation (bending or fanning axons) [23]. This meta-analysis demonstrates a significant reduction of intracellular signal fraction in the NAWM of MS subjects compared with the WM of controls, suggesting early axonal pathology outside demyelination in WM lesions [30,31,32,33]. This consistent reduction among all studies proves that the NDI (through its sensitivity to the intra-axonal compartment) may quantify and differentiate microstructural changes in WM MS lesions, in the NAWM of MS, and in healthy WM. This suggests a loss of axonal density in WM lesions and NAWM in MS compared with healthy controls, which is consistent with previous DTI studies [10,34] reporting altered water diffusion in NAWM and WM MS lesions compared with controlled subjects. Interestingly, the reduction of the NDI in WM lesions and NAWM is in line with previous pathological studies showing axonal loss within WM lesions and a lesser degree in NAWM [17,33,34,35]. This consistent reduction of the NDI reported in this meta-analysis may further assist researchers in relying on NODDI metrics in biophysically understanding the axonal loss/damage in WM lesions and NAWM.

Few studies reported a reduced ODI within the WM lesions than in healthy WM, suggesting that neurites have less orientation variability or loss of neuronal fibres, which can be more severe in lesions [20,24,25]. In contrast, the higher ODI within the WM lesions and NAWM, more than in healthy WM, might suggest a loss of fibre coherence and relatively maintained neuronal fibre density [20,24,25,26]. However, our meta-analysed results contradict these reported results, showing no differences in the orientation dispersion of neurites in patients with MS. In addition, the ODI may vary across regions of the brain and can be very diverse across MS subjects, depending on the stage of MS. Therefore, the ODI may be extracted from heterogeneous areas of interest. Most importantly, the variability of b-values/scanning protocols may lead to heterogeneity and influence the ODI findings of this meta-analysis. For this reason, prospective researchers in the field may consider that NODDI metrics require careful interpretation and a study/scanning protocol design. There are some limitations to this systematic review. First, the NDI and ODI from the ultra-high field MRI have not been pooled due to a limited number of studies. Second, the isotropic signal fraction (isoVF) was not included and pooled because it has only been investigated in two studies [20,24]. Third, the few studies included in this review may lead to insufficient conflicting ODI results, suggesting that the ODI is a less robust biomarker of MS microstructural damage and requires more studies and careful assessment. Fourth, this review lacks the evaluation of neurite density and orientation in grey matter and cortical lesions, or between WM lesions and NAWM. Fifth, the NODDI model is based on several assumptions that may be violated in abnormal tissue structures, leading to a few limitations in the interpretation of results. The T2 relaxation effect that modulates the diffusion MRI signal is not modelled in NODDI. Therefore, any alteration in the T2 relaxation times of the tissue compartments may affect the NODDI parameters. In addition, NODDI assumes a fixed parallel diffusivity for intra-axonal and extra-axonal compartments. If this parameter increases within a WM lesion, the estimated NODDI parameters will be biased. The final limitation is that interpreting ODI alterations is challenging and dependant on the examined ROI. For instance, axonal loss in single or multiple fibre WM regions may increase or decrease ODI values. Therefore, knowing the local fibre structure is important to interpret the ODI correctly. Sixth, there is moderate to considerable heterogeneity between studies, suggesting publication bias between studies that reported the NDI in WM lesions; however, no publication bias was found among studies that reported the NDI in NAWM. In addition, data for WM lesions and NAWM are derived largely from the same publications, thus making publication bias an unlikely explanation for the heterogeneity. This heterogeneity between the included studies possibly arises from the methodology, scanning protocol, data acquisition, and hardware variations. Importantly, the heterogeneity may be explained by the known lack of pathological specificity of the WM lesions. Finally, data on the effect of the disease-modifying treatment on NODDI measures are lacking.

## 5. Conclusions

This systematic review and meta-analysis confirmed that the NDI is significantly reduced in MS lesions and NAWM than WM from healthy participants, which corresponds to the reduced intracellular signal fraction and may reflect underlying damage or loss of neurites. We were unable to demonstrate differences in the ODI in MS lesions and NAWM compared to WM from healthy participants but have identified that heterogeneity in studies may limit the meta-analysis. Further analysis of the NODDI approach in MS for the characterisation of disease-related ultrastructural changes is justified.

## Figures and Tables

**Table 1:** Demographics and clinical characteristics of the included NODDI studies on MS. Abbreviations: NA = not available; HC = healthy controls; * = mean and standard deviation; -- = not applicable; SD = standard deviation; RRMS = relapsing remitting multiple sclerosis; PPMS = primary progressive multiple sclerosis; and CIS = clinically isolated syndromes.

Demographic and Clinical Characteristics of MS Patients and Healthy Individuals							
Study	Author	Population Group	Sample Size	Gender (Number of Females)	Age (Mean ± SD)	Disease Duration Year (Mean ± SD)	EDSS (Median (Range))
1	Collorone, 2019 [27]	RRMS	28	23	39.4 ± 6.6	8 ± 5.6	2.5 (1–6.5)
		HC	20	13	36.6 ± 12.5	--	--
2	De Santis, 2019 [25]	RRMS	7	7/NA	42 ± 15	21 ± 11	1 (0–3)
		HC	6	6/NA	42 ± 15	--	--
3	Granberg, 2017 [23]	RRMS	26	21	39.0 ± 8.2	2.5 ± 1.4	1.5 (0–4)
		HC	24	17	37.7 ± 10.6	--	--
4	Hagiwara, 2019 [24]	RRMS	24	19	39.83 ± 8.25	11.82 ± 5.99	1 (0–7)
		HC	24	19	39.50 ± 11.13	--	--
5	Rahmanzadeh, 2021 [29]	RRMS & PPMS	91	56	46 ± 14	NA	2.5 (0–8)
		HC	72	43	36 ± 12		--
6	Sacco, 2020 [26]	RRMS, CIS, PPMS	21	17	36.4 ± 8.7	NA	* 2.6 ± 1.6
		HC	21	17	36.4 ± 8.7		
7	Schneider, 2017 [20]	RRMS	5	3	39.2 ± 8.6	11 ± 3.4	4 (3–6)
		HC	5	3	37.6 ± 12.3	--	--

**Table 2:** Technical characteristics of included studies. Abbreviations: NA = not available; T = tesla; ROI = region of interest; NAWM = normal-appearing white matter; DAWM = dirty-appearing white matter; NAGM = normal-appearing grey matter; WM = white matter; RF = radiofrequency; and s·mm^−2^ = second per millimetre.

Technical Characteristics of the Included Studies					
Study	Author	Field Strength	RF Coil/ Fitting Toolbox	b-Values (s·mm^−2^)	Method of Analysis/Regions
1	Collorone, 2020 [27]	3.0 T	32 NODDI MATLAB	300–1000–2855	ROI: NAWM
2	De Santis, 2019 [25]	3.0 T 7.0 T	NA MDT	700–2000	ROI: WM lesions, NAWM, and NAGM
3	Granberg, 2017 [23]	3.0 T	64 NODDI MATLAB	1000–5000	ROI: cortical lesions, WM lesions, and NAWM
4	Hagiwara, 2019 [24]	3.0 T	19 (AMICO)	1000–2000	Voxel wise whole brain: WM
					ROI: NAWM and WM lesions
5	Rahmanzadeh, 2021 [29]	3.0 T	64 (AMICO)	700–1000–2000–3000	ROI: WM lesions, NAWM, and NAGM
6	Sacco, 2020 [26]	3.0 T	NA NODDI MATLAB	700–2000	ROI: WM lesions, NAWM, and DAWM
7	Schneider, 2017 [20]	3.0 T	32 NODDI MATLAB	300–711–2000	ROI: WM, NAWM, and WM lesions

## Data Availability

No new data is created or analysed in this study. Data sharing is not applicable to this article.
